# Prevalence and tracking of back pain from childhood to adolescence

**DOI:** 10.1186/1471-2474-12-98

**Published:** 2011-05-16

**Authors:** Per Kjaer, Niels Wedderkopp, Lars Korsholm, Charlotte Leboeuf-Yde

**Affiliations:** 1 Institute of Sports Science and Clinical Biomechanics, Part of Clinical Locomotion Network, University of Southern Denmark, Campusvej 55, DK-5230, Odense, Denmark; 2 Research Department, Spine Centre of Southern Denmark, Part of Clinical Locomotion Network, Hospital Lillebaelt, Institute of Regional Health Research University of Southern Denmark, Ostre Hougvej 55, DK-5500 Middelfart, Denmark; 3 Nycomed Danmark, Langebjerg 1, DK-4000 Roskilde, Denmark

## Abstract

**Background:**

It is generally acknowledged that back pain (BP) is a common condition already in childhood. However, the development until early adulthood is not well understood and, in particular, not the individual tracking pattern. The objectives of this paper are to show the prevalence estimates of BP, low back pain (LBP), mid back pain (MBP), neck pain (NP), and care-seeking because of BP at three different ages (9, 13 and15 years) and how the BP reporting tracks over these age groups over three consecutive surveys.

**Methods:**

A longitudinal cohort study was carried out from the years of 1997 till 2005, collecting interview data from children who were sampled to be representative of Danish schoolchildren. BP was defined overall and specifically in the three spinal regions as having reported pain within the past month. The prevalence estimates and the various patterns of BP reporting over time are presented as percentages.

**Results:**

Of the 771 children sampled, 62%, 57%, and 58% participated in the three back surveys and 34% participated in all three. The prevalence estimates for children at the ages of 9, 13, and 15, respectively, were for BP 33%, 28%, and 48%; for LBP 4%, 22%, and 36%; for MBP 20%, 13%, and 35%; and for NP 10%, 7%, and 15%. Seeking care for BP increased from 6% and 8% at the two youngest ages to 34% at the oldest. Only 7% of the children who participated in all three surveys reported BP each time and 30% of these always reported no pain. The patterns of development differed for the three spinal regions and between genders. Status at the previous survey predicted status at the next survey, so that those who had pain before were more likely to report pain again and vice versa. This was most pronounced for care-seeking.

**Conclusion:**

It was confirmed that BP starts early in life, but the patterns of onset and development over time vary for different parts of the spine and between genders. Because of these differences, it is recommended to report on BP in youngsters separately for the three spinal regions, and to differentiate in the analyses between the genders and age groups. Although only a small minority reported BP at two or all three surveys, tracking of BP (particularly NP) and care seeking was noted from one survey to the other. On the positive side, individuals without BP at a previous survey were likely to remain pain free at the subsequent survey.

## Background

It is well known that back pain (BP) is a common and costly problem in the general population. Previously, BP in children was considered rare and a sign of a potentially serious disorder [1,2]. Today, according to a recent systematic review, the general opinion would be that BP, including low back pain (LBP), mid back pain (MBP) and neck pain (NP), starts already early in life to accelerate during the early teens up till early adulthood [3] and that its presence in young age is a precursor for BP also in adulthood [4]. In order to approach the issues of prevention and treatment it is helpful to understand the extent and course of a disease, particularly around the time of its onset and that picture is, presently, far from clear. Methodological and definition issues can partly explain this [3]. However, this is also a question of the study objectives and design. It is therefore not surprising that the estimates from various studies vary and that often they make no sense. Also, there appears to be no credible data on the true incidence for each spinal region in young people.

In addition, it is not clear to what extent BP in youngsters results in consequences such as those seen in adults, namely reduced activities, sick leave (i.e. absence from school), and consultations with health care practitioners. We found only few studies on children and adolescents dealing with this topic but their conclusions differed. Auvinen et al [5] reported that the seeking of health care increased with age for both LBP and NP, whereas others found no such increase [6,7]. In another study, no associations with age were found for reduced activities and taking time off from school [8]. Others who reported on this issue did not take age into account. It is therefore not known if these consequences are proportional to the prevalence of pain at the various ages or if the consequences have an age-related profile of their own.

Theoretically, the prevalence rates in cohorts born at different times could be affected by dissimilar living conditions rather than by being a product of age. Therefore, in order to study the development over time in individuals, it would be more correct to follow a cohort over time rather than comparing prevalence estimates for children of different age groups, who were all surveyed at the same time. Although population-based studies have been published on the trajectories of back pain in general over time in young people [9,10], we found no study in which all three spinal regions had been investigated prospectively. Because the onset of pain in the three different spinal regions previously was shown to arise at different ages [11] and because of the obviously different anatomical and biomechanical properties of the lumbar, thoracic and cervical regions, we considered it relevant to study these spinal regions independently.

In order to obtain more information in this area we collated data obtained in three previous studies on back pain in children, who were considered representative of the general Danish population. The two main objectives of our study were:

1. To describe the prevalence of BP (including LBP, MB and NP) and care seeking in these children when they were aged 9, 13 and 15 years.

2. To study to which extent BP and care seeking track over time in these children at these three points in time.

In addition, we reported data for boys and girls separately, and took into account the tracking pattern also for those who failed to participate in the previous survey.

## Methods

### Study design

The prevalence estimates of BP and care seeking are presented by age and gender based on three cross-sectional studies of a cohort of children studied at the ages of 9, 13 and 15 years. The tracking of BP and care seeking is based on the individual trajectories over time. The three studies are referred to as T1, T2, and T3, where "T" stands for "time".

### The flow of the study

The original cohort was sampled for the European Youth Heart Study (EYHS) in 1997 among 3^rd ^grade schoolchildren in the municipality of Odense, Denmark. The main purpose of that study was to investigate risk factors for cardiovascular disease and diabetes in a mixed longitudinal study design. As a part of that study, an interview was conducted to determine whether back problems were present. All interviews at T1 were performed at the schools of the children [8] but due to logistic problems, 110 of the sampled children were not then offered a back interview.

Four years later, in 2001/2, those participating in the study at T1 and still living in the same area were invited to a second investigation regarding BP. At this second time point the children had an MRI of the lumbar spine, were questioned about BP, and measurements of body composition were taken [12]. All children were picked up at their schools by taxi and brought to the Spine Centre of Southern Denmark, Ringe.

**Figure 1:**
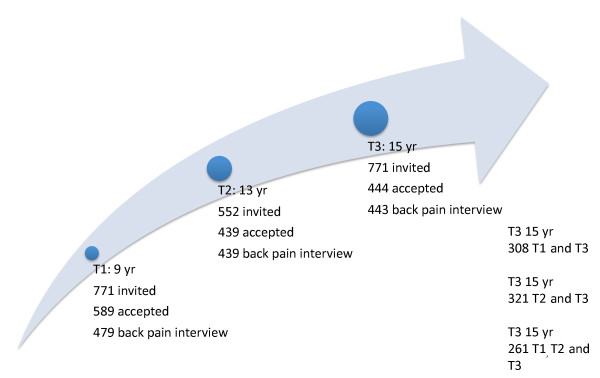
**Flowchart**. Flow of participants in a longitudinal study of Danish children/adolescents at three time points (T1, T2 and T3).

The third data collection took place in 2003/2004. At this time, all teenagers sampled for the original cohort were invited to an interview about BP and its consequences. They also had an MRI of the lumbar spine. Furthermore, a number of objective measurements were taken for back performance, body composition, and aerobic capacity. In addition, physical activity was measured objectively over a one-week period. Also this data collection took place at the Spine Centre of Southern Denmark. The children were brought to the centre by taxi or by train, if they had moved to other parts of the country. The flow of participants is shown in Figure 1.

### Study population

The 38 relevant state schools in the municipality of Odense (180,000 inhabitants) were stratified according to their location (urban, suburban, rural) and the socio-economic character of their uptake area [8]. From each stratum, a proportional, two-stage cluster sample of children was selected. The primary units (clusters) were the schools. The sampling frame was all the schools in the town, from which schools were selected using probability, proportional to school size. Each school on the list was allocated a weighting equivalent to the number of children enrolled, who were eligible to be selected for the study. The secondary units were the children in the schools. Equal numbers of children in the third and ninth grades (a maximum of 30 individuals from each grade and school) were sampled from each school. Children in the appropriate age bands (8 to 10 years and 14 to 16 years) were allocated code numbers and then randomly selected using random number tables. For this report, only the young cohort is used.

Experience from previous studies of children using a similar method suggested that a likely response rate from schools would be 90%, and that a 75% to 80% response rate from the children or their parents could be expected [13,14]. Estimates of power were based on the cardiovascular aspect of the study, which is described elsewhere, and a maximum non-response rate of 25% was predicted [11]. This required a minimum of 4 × 250 participants (i.e. 250 children in each age and gender subgroup).

### Generalizability

It has been shown that the income of the parents of the children participating at T1 was similar to the rest of the Danish general population, whereas the parents' educational level was slightly higher [15]. We have not further addressed the characteristics of non-responders.

### Ethics

The study was approved by the local ethics committee (ref. no. 20000042) and the database was approved the Danish Data Protection Agency (ref. no. 2000-53-0037). The children as well as their parents gave their consent to participate in the study

### Data collection

Data on LBP were collected using an interview developed on the basis of previously used nation-wide surveys and tested for feasibility in relation to the first data collection [8,16]. The children were asked if they had any spinal pain (LBP, MBP or NP) at the moment, within the past week, or within the past month in order to establish the one-month period prevalence. Those who responded positively to any of these questions were asked to show the location of the pain. The lumbo-pelvic, thoracic, and cervical spine and the corresponding posterior aspects of the body surface were defined as low back, middle back, and neck, respectively. If the child had problems showing the area, the interviewer put one hand subsequently on the neck, thoracic and lumbar area while asking: "Was it in this area?"

If the child reported back pain, the following questions were asked: "Did you because of back pain a) stop participation in physical activity such as sports or play? b) stay home from school up to 3 days, c) stay home from school for more than 3 days? d) see a physician once? e) see a physician more than once? f) see a physical therapist or participate in special gymnastics? g) go to a hospital?" The question f) "see a physical therapist" was included in the second and third surveys only [12].

At T1 the interview took place at the schools and was conducted by NW. All questions were explained to ensure the children's understanding of the content of the questions. The same procedure was used at T2 and T3. At T2 the interview took place in relation to the MRI scanning and was conducted by either of two radiographers and at T3 a research nurse conducted the interview.

### Validity

At T2 the children also filled in a questionnaire on back pain that had previously been used on Danish schoolchildren [17]. Therefore, the possibility of comparing answers from interviews with answers from questionnaire existed. Analyses of the different responses lead to the conclusion that data from the interview were the most credible [18].

### Data manipulation

From the interview, "BP" was recorded if the child reported pain in any of the spinal areas on the day of examination, within the past week, or within the past month. "LBP" was defined as positive if the child answered yes to pain related to the lumbar area, "MBP" for pain related to the thoracic spine, and "NP" for pain related to the cervical area. "Seeking care" was defined as such if "yes" was the answer to any of the questions d-g about consequences of back pain.

### Quality of data

Data from the interviews were collected on paper and entered into data files using the software Microsoft Access for the first study. All computerised data were double checked against the original data on paper, and corrected if necessary. Data from the second round were entered into the software database EpiData and were checked randomly with an extremely low error rate. Information from the third data collection was entered directly into EpiData, leaving no possibilities for missing data. If the child could not provide a lucid answer, the response was nevertheless entered in a log-file for future decision/classification.

### Statistical analyses

Prevalence data were reported for each variable in each cross-sectional study. Exact 95% confidence intervals were constructed and differences in proportions between genders tested with Fisher's exact test. Test for trend over time was performed using logistic regression accounting for repeated measures by Stata's cluster option in order to define statistically significant differences in prevalence rates between the surveys. The tracking patterns of BP, LBP, MBP, NP, and care seeking were studied by investigating how study subjects stayed in or left their respective group when surveyed at the next time point. The patterns of reporting at the second and third surveys were also established for previous non-responders in order to see if they were biased towards more BP than the responders.

## Results

In all, 62% of the 771 children, who were invited and eligible to participate in the study, participated at the age of 9. At the age of 13, 57% participated and the percentage was 58 at the age of 15. Thirty-four percent of pupils participated in all three surveys, 41% at T2 and T3, and 42% at T1 and T3. For detailed flow over the three studies see Figure 1 and for information on gender, age, time between interviews and period of data collections see Table 1.

**Table 1:** Summary of demographic and study data in a longitudinal study of Danish children/adolescents at three time points (T1, T2 and T3).

Description of studies			
**Time point**	**T1**	**T2**	**T3**

Number of participants: males/females	227/252	250/234	192/251
Age mean [range]	9.7 [8.7,15.5]	13.1 [12.1 - 14.4]	15.7 [14.7 - 17.1]
Years since last interview [range]		3.4 [3.0 - 4.0]	2.7 [2.2 - 3.1]
Period of data collection	August 1997 -June 1998	March 2001 -October 2001	November 2003 -May 2004

### The prevalence of back pain and seeking care

The prevalence estimates of BP, LBP, MBP and NP at the different surveys have been reported as percentages for all and separately for boys and girls, with some of the differences being statistically significant (see Table 2). The prevalence of BP was 33% at T1 and 48% at T3. The widest gap was noted for LBP, which went from 4% to 35%. Mostly, the estimates were higher for girls. It was rare (6%) to report having sought care at the two first surveys whereas it was almost 6 times more common (34%) at the age of 15.

**Table 2:** Prevalence rates of different types of back pain and care seeking in a cohort of Danish children/adolescents surveyed at three time points (T1, T2 and T3).

Prevalence of back pain and care seeking									
	**T1 9 yr**			**T2 13 yr**			**T3 15 yr**		

	N	n	(%)	N	n	(%)	N	n	(%)
Backpain									
all	479	159	(33)	439	124	(28)	443	211	(48)
boys	227	81	(32)	205	53	(26)	192	75	(39)
girls	252	78	(34)	234	71	(30)	251	136	(54)**^1^**
LBP									
all	479	21	(4)	439	98	(22)	443	157	(35)
boys	227	15	(7)	205	38	(19)	192	49	(26)
girls	252	6	(2)**^2^**	234	60	(26)	251	108	(43)**^3^**
Mid back pain									
all	479	97	(20)	439	57	(13)	443	122	(28)
boys	227	49	(22)	205	26	(13)	192	42	(22)
girls	252	48	(19)	234	31	(13)	251	18	(32)**^3^**
Neck pain									
all	482	49	(10)	439	31	(7)	443	68	(15)
boys	227	21	(9)	205	11	(5)	192	24	(13)
girls	252	28	(11)	234	20	(9)	251	44	(18)
Seeking care for back pain									
all	479	30	(6)	439	34	(8)	443	150	(34)
boys	227	15	(7)	205	14	(7)	192	59	(31)
girls	252	15	(6)	234	20	(9)	251	91	(36)

N refers to the total number of children and n the number of children with a certain type of back pain.

^1 ^p = 0.002, ^2 ^p = 0.027, and ^3 ^p < 0.000. Fishers exact test

**Figure 2:**
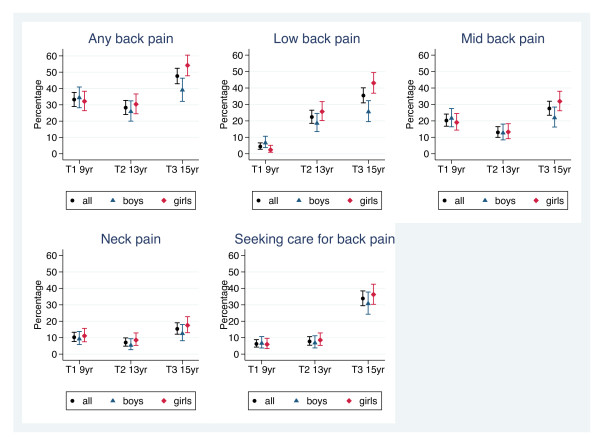
**Prevalence of back pain and seeking care**. The one month prevalence rates given in percentages for back, neck, mid back, and low back pain as well as related care seeking in a cohort of Danish children/adolescents at three time points (T1 (N = 479), T2 (N = 439), and T3 (N = 443). The bars indicate the 95% confidence intervals for the estimates.

The reporting of BP accelerates after the age of 13 and even more for seeking care for BP. The acceleration seen in BP is mainly explained by the increasing prevalence of LBP, which in fact is seen already at 13. For detailed information, see Table 2 and for an overview Figure 2. At the age of 9, MBP is most commonly reported, whereas LBP is most common at the ages of 13 and 15. Although the test for trend showed statistically significant increases over time (p values ranging from p < 0.000 to p = 0.038), MBP and NP seem to have different profiles to that of LBP, with a "dip" at T2, whereas LBP increases in a step-wise fashion (Figure 2).

### Tracking of back pain and seeking care

Regardless whether the children reported pain or no pain in a specific spinal area, they were more likely to report no pain than pain in that region in the subsequent survey (Table 3 columns 2 and 3 show that the proportions reporting pain are lower than 50%). Nevertheless, having reported pain in one survey (as compared to not having reported pain) increases the probability of reporting pain again in the next survey and this finding is markedly higher between T2 and T3 (Table 3 columns 2 and 3). This finding was most pronounced in the case of seeking care, with almost 90% of those who reported to have sought care at T2 doing so again at T3, whereas only 30% who reported no care-seeking at T2 reversed into care-seeking at T3 (Table 3 column 3, rows 13 and 14). A similarly pronounced tracking pattern was found for NP (Table 3 column 3, rows 10 and 11) with 40% vs. 13% reporting NP at T3. As a consequence, tracking went also in the opposite direction, meaning that absence of pain predicted continued absence of pain.

**Table 3:** Probability (given in percentage) of reporting back pain depending on the back pain status at the previous data collection point in a cohort of Danish children/adolescents surveyed at three time points (T1, T2 and T3).

Tracking of back pain and seeking care		
**Status**	**% with pain T2** **depending on status at T1**	**% with pain T3** **depending on status at T2**

*BP*	33	58
No BP	26	46
Not in study	27	42
*LBP*	32	38
No LBP	21	34
Not in study	24	30
*MBP*	18	47
No MBP	11	26
Not in study	14	24
*NP*	11	40
No NP	7	13
Not in study	4	17
*Seeking care for BP*	17	**88**
Not seeking care	7	30
Not in study	9	30

Those who were present at one survey but absent at the preceding survey were never most likely to be categorised as having pain (or seeking care) at the subsequent survey (Table 3 "not in study", 2^nd ^and 3^rd ^columns). In fact, at the time of T3, those who did not take part in the second survey were the least likely to report spinal pain (Table 3 "not in study", 3^rd ^column).

In all, 7% of the children who participated in all three surveys (n = 261) reported to have had BP at all three surveys and 30% always reported no BP. The same percentages were < 1% vs. 49% for LBP, 1% vs. 60% for MBP, < 1% vs. 71% for NP, and 1% vs. 60% for seeking care for back pain.

**Figure 3:**
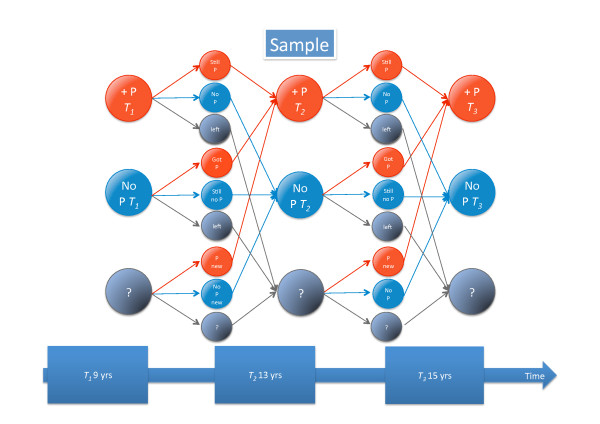
**Explanation of Figures 4-8**. Patterns of reporting types of back pain or seeking care for back pain. P = pain. Red is pain reporting, blue, no pain reporting, and grey is those not in study. The arrows to the smaller circles show the patterns of reporting at the next time-point: still P (reporting pain again), no P (now not reporting pain), left (leaving the study), Got P (changing from not reporting to reporting pain), Still no P (reporting no pain again). P new (entering the study and reporting pain, No P new (entering the study but not reporting pain), question marks (not in study at that specific time of data collection). The three small circles do not sum to 100%: The percentages with pain and without pain for those still in study are given whereas the percentage of those leaving or staying out of the study are given in relation to the number of people in that category in previous study.

**Figure 4:**
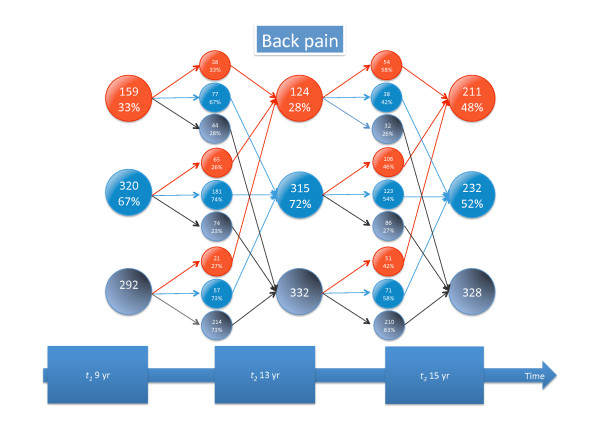
**Back pain tracking**. For explanation, see Figure 3.

**Figure 5:**
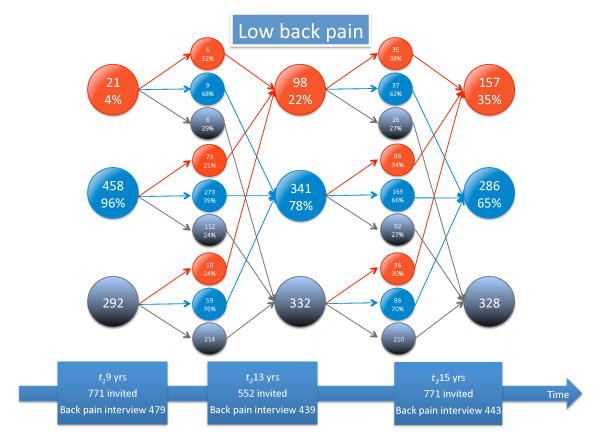
**Low back pain tracking**. For explanation, see Figure 3.

**Figure 6:**
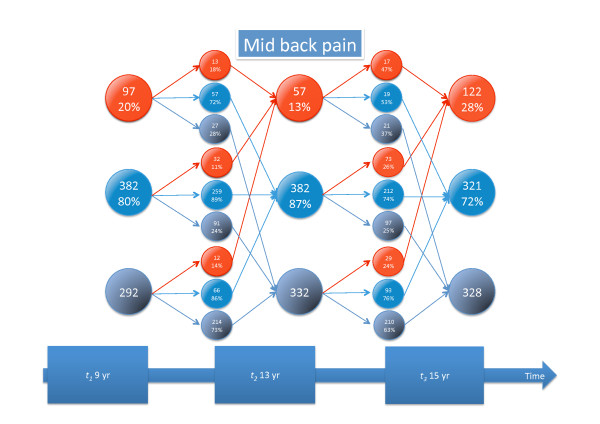
**Mid back pain tracking**. For explanation, see Figure 3.

**Figure 7:**
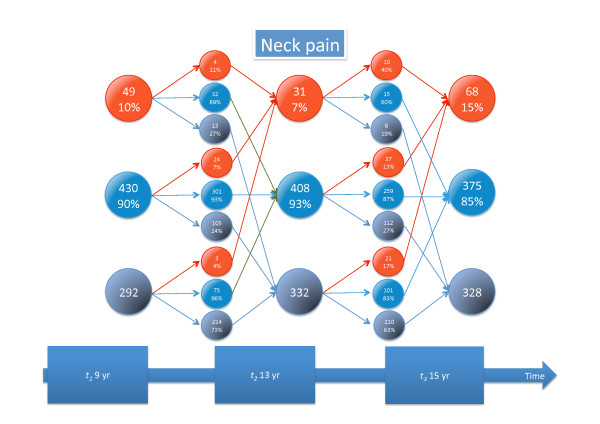
**Neck pain tracking**. For explanation, see Figure 3.

**Figure 8:**
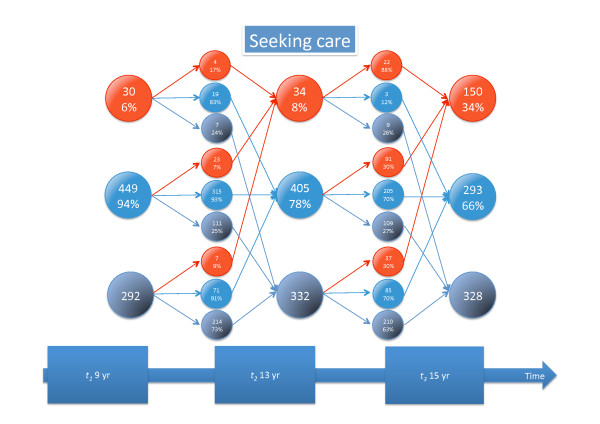
**Tracking of seeking care for back pain**. For explanation, see Figure 3.

Detailed information about tracking of different types of back pain and seeking care is given in Figures 3, 4, 5, 6, 7, and 8.

## Discussion

### Summary of findings

This is to our knowledge the first study to report BP, LBP, MBP and NP at three different points in time over a 6-year period in one cohort of children/adolescents. Overall, absence of pain was the most common finding. When considering those with pain, it was noted that in the youngest group (9 yrs), MBP was the most common complaint, whereas LBP was most commonly reported at the next two surveys. At the youngest age, boys reported most LBP, MBP, and seeking care for BP but were overtaken by the girls already at 13. Care seeking was very uncommon in the youngest group and much less common than BP (6% vs. 33%). At the age of 15, this gap had decreased (34% vs. 48%), indicating either that the symptoms are now more bothersome or that the pain is not taken seriously by the parents until the child is older.

A rapid increase in LBP reporting was seen from the age of nine to 13 and it was, in particular, those who previously had pain who were more likely to report it again. For the last survey this was noted to be very pronounced in some variables (previous care seeking and previous NP). It was somewhat less marked for MBP and BP in general, whereas this finding was almost absent for LBP. The findings in the second survey were less evident. Of the 261 children who participated in all three surveys, only 7% reported BP all three times and 30% reported no pain all three times, showing that frequent or constant pain in this age group is not yet common.

Comparisons with other studies are not easily done, as we could not find any that included the same age groups and studied all spinal areas. There is, of course, no obvious reason to doubt that BP starts early and since it is more common in young adults than in children, it has to increase in adolescence. This has been shown, for example, by Stanford et al in a recent population-based study on "weekly or more recurrent BP" [10]. That BP at this age is fairly uncommon was convincingly shown also in a study by Dunn et al [9]. About 3/4 of their 11-14 yr olds belonged to the "no pain problem" groups, when BP was defined as "pain in the past three months that lasted a whole day or more, or that had occurred several times in a year" and when the trajectory pattern was measured as often as three times per year over three years. The stability of this pattern (tracking) was apparent also from their results.

### Strength and weaknesses of the study

A strength of this study is that the study sample was taken from the general population and can be considered relatively representative of the general Danish population. Nevertheless, because the majority of children failed to participate at all three surveys, it was important to study the pain pattern in the "sometimes" participants. One could expect that the more disadvantaged children would be those who more likely to abstain from participation, and that they might be more likely to have BP. However, the non-participants who appeared either in the second or third survey were seen to be more likely to have no BP or, at least, not to feel concerned about BP. The reason for this is perhaps that some of the "previous" non-participants were rather uninterested in the study, precisely because they had no BP. On the other hand, those who had BP might have been more interested in participating, as participation in the study meant access to a free MRI of the lumbar spine. An over representation of participants with BP would obviously increase the prevalence estimates and affect the tracking results, but the overall picture that emerges from this study appears clear, despite this.

Retrospective self-reported BP estimates in both adults and children are probably rather approximative. It is therefore important that the child who participates in a study clearly understands what is meant by "BP". In these surveys we used a method previously shown to give credible results [11] and the interview was performed by one person in the first and the last survey whereas two radiographers took turns at the second survey. This is likely to provide credible results. Also, the length of the recall period was such as to optimize the validity of this variable. Intuitively, a one-month recall period would be acceptable, particularly as several questions were asked in relation to BP: "today", "last week" and "in the past month", increasing the children's possibility to recall past events.

## Conclusion

It was confirmed that BP starts early in life, but the patterns of onset and development over time vary for different parts of the spine and between genders. Because of these differences, it is recommended to report on BP in youngsters separately for the three spinal regions, and to differentiate in the analyses between the genders and age groups. Although only a small minority reported BP at two or all three surveys, tracking of BP (particularly NP) and care seeking was noted from one survey to the other. On the positive side, individuals without BP at a previous survey were likely to remain pain free at the subsequent survey.

## Abbreviations

BP: back pain; LBP: low back pain; MBP: mid back pain; NP: neck pain; EYHS: European Youth Hearth Study; T1: Time point 1; T2: Time point 2; T3: Time point 3;

## Competing interests

The authors declare that they have no competing interests.

## Authors' contributions

NW was responsible in establishing the EYHS study, sampled the initial cohort and collected data at T1, assisted in drafting the manuscript, analyzing and interpreting the results, and critically revised the manuscript. PK participated in the development of concept and design, collected data at T2 and T3, analysed the data, drafted the methods and results, and did the final revisions of the manuscript.

LK was involved in the concept and design of the study, supervised the analyses, participated interpretation of the results, and critically revised the manuscript.

CLY took part in the development of concept and design, data-management, analyses and -interpretation, drafted the background and discussion. She also critically revised the manuscript. All authors read and approved the final version of the manuscript.

## Pre-publication history

The pre-publication history for this paper can be accessed here:

http://www.biomedcentral.com/1471-2474/12/98/prepub
